# Advancing rehabilitation in Parkinson’s disease through virtual reality: a narrative review

**DOI:** 10.3389/fneur.2026.1761459

**Published:** 2026-05-14

**Authors:** Pierluigi Diotaiuti, Francesco Di Siena, Marco Palombo, Giulio Marotta, Elisa Cavicchiolo, Pio Alfredo Di Tore, Stefania Mancone

**Affiliations:** 1 Department of Human Sciences, Society and Health, University of Cassino and Southern Lazio, Cassino, Italy; 2 Department of Systems Medicine, University of Rome Tor Vergata, Rome, Italy

**Keywords:** Parkinson’s disease, virtual reality, neurorehabilitation, motor rehabilitation, gait training, balance training, cognitive rehabilitation, neuroplasticity

## Abstract

**Background:**

Virtual reality (VR) is increasingly explored in Parkinson’s disease (PD) rehabilitation, yet the clinical evidence remains heterogeneous.

**Objective:**

To provide an integrative narrative synthesis of the neurophysiological rationale, VR modalities (immersive, semi-immersive, and non-immersive), and motor/non-motor outcomes associated with VR-based rehabilitation in PD.

**Methods:**

Narrative review of peer-reviewed studies published between 2010 and 2025. Fifteen PD + VR rehabilitation studies meeting eligibility criteria were included in the qualitative synthesis. Mechanistic, non-PD, pre-2010, or otherwise non-eligible studies were discussed only as contextual/supporting evidence. Findings were synthesized narratively, and methodological limitations were examined through a structured critical appraisal.

**Objective:**

Across small and methodologically heterogeneous samples, VR-based rehabilitation was associated mainly with improvements in balance and gait-related outcomes, including Berg Balance Scale (BBS) scores, Timed Up and Go (TUG) performance, Functional Gait Assessment (FGA), stride length, gait velocity, and walking distance. Selected studies also reported gains in upper-limb function and balance confidence. Effects on freezing of gait and tremor were variable. Cognitive and executive effects were less established than motor findings and should be considered preliminary.

**Conclusion:**

VR appears promising as an adjunct to conventional rehabilitation in PD, particularly for gait, balance, and motor-cognitive training, but the certainty of evidence remains moderate to low. Broader mechanistic interpretations should be considered plausible explanatory frameworks rather than demonstrated therapeutic effects. Priorities for future research include protocol standardization, blinded assessment, improved safety reporting, sham/yoked comparator designs, responder profiling, and evaluation of scalable home-based and AI-adaptive platforms.

## 1 Introduction

Parkinson’s Disease (PD) is a progressive neurodegenerative disorder that primarily affects the dopaminergic neurons in the substantia nigra pars compacta, leading to dopamine depletion in the basal ganglia. The loss of dopaminergic transmission disrupts motor control circuits, resulting in cardinal motor symptoms such as bradykinesia (slowness of movement), postural instability, resting tremors, and rigidity. However, PD is not solely a movement disorder; it is increasingly recognized as a multisystem condition affecting both motor and non-motor functions (1–5).

In addition to its well-documented motor impairments, PD is associated with a range of non-motor symptoms, including cognitive dysfunction, executive deficits, visuospatial impairments, autonomic dysregulation, mood disturbances, and sleep disorders (6–11). These non-motor symptoms, particularly cognitive decline and emotional dysregulation, significantly impact quality of life and functional independence. The complex interplay between motor dysfunction, executive deficits, and neuropsychiatric symptoms underscores the need for multimodal therapeutic approaches that target both physical and cognitive impairments in PD.

Traditional rehabilitation approaches, including physical therapy, occupational therapy, and balance training, remain central in Parkinson’s disease (PD) care because they target gait dysfunction, postural instability, and mobility limitations. However, their long-term impact is often constrained by several factors. First, adherence may be suboptimal, as repetitive exercises, transportation barriers, and progressive motor and non-motor symptoms can reduce sustained participation. Second, many conventional programs are delivered in controlled clinical environments that only partially reflect the complexity and unpredictability of everyday mobility, thereby limiting ecological validity and transfer to real-world function. Third, standard rehabilitation often prioritizes motor practice while insufficiently engaging the cognitive and executive processes that strongly influence gait, balance, dual-task performance, and fall risk in PD.

In this context, virtual reality (VR) has emerged as a promising adjunct to neurological rehabilitation. By combining interactive virtual scenarios, real-time multisensory feedback, and task-specific training, VR may help address some limitations of conventional therapy while promoting a more engaging and adaptable rehabilitation environment. In PD, VR-based interventions have been used to simulate walking challenges, balance tasks, upper-limb activities, and cognitive-motor exercises in ways that can increase motivation, support repeated practice, and enable progressive adjustment of task difficulty. These features make VR particularly relevant for interventions aimed at gait, postural control, motor learning, and dual-task integration (12–16).

The rationale for VR in PD rehabilitation is further supported by its potential to deliver augmented feedback, error-based learning, and cognitively enriched motor practice. Through visual, auditory, and proprioceptive cues, VR may facilitate movement correction, postural adaptation, and individualized training progression (16–23). At the same time, its gamified and feedback-rich structure may improve engagement and adherence, two persistent challenges in long-term rehabilitation (24–26). For these reasons, VR has attracted growing interest as a flexible rehabilitation tool capable of addressing both motor and selected cognitive dimensions of PD, although the clinical evidence remains heterogeneous and methodologically mixed.

One of the most extensively studied applications of VR in PD is gait and balance training, as postural instability and gait disturbances significantly contribute to reduced mobility and fall-related injuries in individuals with PD. VR-enhanced gait training programs incorporate visual and auditory feedback, obstacle navigation challenges, and real-time step correction, helping patients improve step length, postural control, and dynamic stability. Studies have demonstrated that VR-based gait interventions can enhance weight shifting, increase cadence, and reduce gait variability, allowing patients to practice real-world walking scenarios in a controlled, fall-safe environment. VR modifies sensory processing during movement, reinforcing motor adaptation and compensatory strategies that improve functional ambulation and reduce fall risk. By integrating dual-task walking exercises, where patients must navigate virtual environments while simultaneously engaging in cognitive tasks, VR also enhances motor-cognitive interactions, which are crucial for maintaining mobility in daily life (27, 28). VR-based rehabilitation has also proven effective in motor learning and upper-limb coordination training, addressing the fine and gross motor impairments that affect many PD patients. VR systems provide task-specific, repetitive motor training, which is essential for motor skill acquisition and retention in PD (29–31). Through gesture-controlled simulations and adaptive motor tasks, patients engage in reach-and-grasp exercises, bimanual coordination training, and precision-based motor tasks designed to improve upper-extremity dexterity and reduce bradykinesia. The integration of real-time kinematic feedback and progressive difficulty levels enhances error correction and neuromuscular control, supporting motor circuit reorganization and plasticity-driven recovery. Compared to traditional fine motor exercises, VR-based interventions offer greater engagement and motivation, encouraging higher repetition rates and longer therapy durations, which are critical for improving movement fluency and coordination.

Beyond its motor rehabilitation applications, VR has demonstrated significant potential in cognitive rehabilitation for PD, addressing the executive function, attentional control, and memory impairments commonly observed in the disease. VR-based cognitive training programs immerse patients in interactive, problem-solving scenarios, where they must navigate virtual environments, complete memory-based tasks, or respond to dynamic stimuli (32–35). These interventions are particularly valuable in enhancing visuospatial processing, working memory, and decision-making skills, which are often compromised in PD. Unlike traditional cognitive training methods, VR introduces multisensory integration, engaging patients in simulated real-world challenges that mimic daily decision-making and problem-solving situations. Research has shown that VR-based cognitive exercises can lead to improvements in reaction times, cognitive flexibility, and attentional shifts, which directly impact functional independence and quality of life in PD patients.

The integration of VR into PD rehabilitation offers a unique opportunity to combine motor and cognitive training in a way that is personalized, adaptable, and engaging. By leveraging real-time feedback, gamified task structures, and immersive sensory engagement, VR fosters higher patient motivation and adherence, addressing a major limitation of conventional rehabilitation programs. As VR technology continues to advance, incorporating artificial intelligence-driven adaptation, biometric feedback, and tele-rehabilitation features, its role in enhancing neuroplasticity, improving functional mobility, and supporting cognitive resilience in PD patients is likely to expand its relevance within future rehabilitation strategies, although its precise clinical role remains to be established through more methodologically robust studies.

Given the increasing research interest in VR-based rehabilitation for PD, this review aims to provide a comprehensive analysis of the current state of VR applications in PD rehabilitation by integrating findings from neuroscientific, clinical, and technological perspectives. The first objective of this review is to examine the neurophysiological mechanisms underlying VR training in PD rehabilitation, exploring how VR-induced sensorimotor stimulation, real-time feedback, and cognitive-motor integration facilitate neuroplasticity and functional recovery. Understanding these mechanisms is essential for optimizing VR intervention design and ensuring that rehabilitation protocols are aligned with the specific neurofunctional deficits observed in PD.

The second objective is to assess the effectiveness of VR-based interventions in improving gait, balance, motor function, and cognitive performance in PD patients. Given the profound impact of gait disturbances, postural instability, and upper-limb coordination deficits on mobility and daily functioning, this review evaluates how VR-based rehabilitation compares to traditional physiotherapy in promoting motor adaptation, fall prevention, and dual-task training.

While VR shows encouraging associations with motor and cognitive outcomes, methodological heterogeneity and small samples preclude firm conclusions on efficacy; this review therefore adopts a cautious, narrative synthesis.

## 2 Methods

### 2.1 Review design and reporting standards

**Figure 1:**
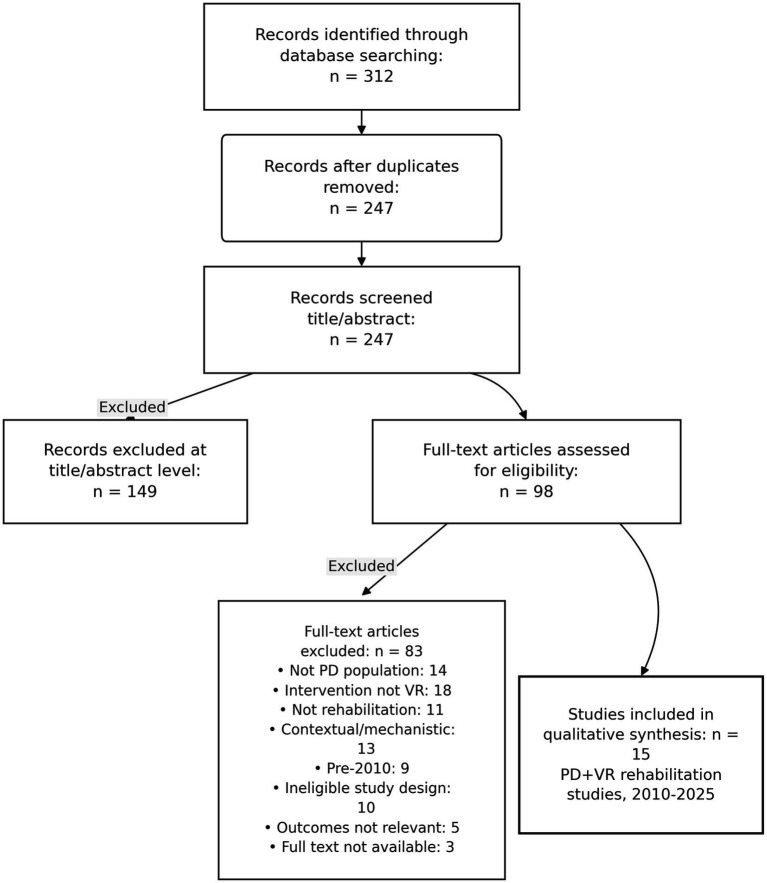
Narrative search flow and study selection (2010–2025).

This work is an Integrative (Narrative) Review providing a qualitative synthesis of clinical evidence on virtual reality (VR)-based rehabilitation in Parkinson’s disease (PD). An integrative approach was chosen due to substantial heterogeneity across protocols (immersion level, dosage, comparators, outcomes) and typically small sample sizes, which preclude a robust meta-analysis. Conduct and reporting follow good-practice criteria for narrative reviews (e.g., SANRA). We include a narrative search flow diagram (Figure 1).

### 2.2 Information sources and search strategy

We searched PubMed, Scopus, Web of Science, and Google Scholar. The final search date was 9 November 2025. The time window covered studies published between 1 January 2010 and 9 November 2025. Search strings combined pathology, intervention, and outcomes, for example: Parkinson* AND (virtual reality OR VR OR head-mounted display OR HMD OR immersive OR semi-immersive OR exergam*); Parkinson* AND (rehabilitation OR physiotherapy) AND (gait OR balance OR freezing OR upper-limb OR cognition); (Kinect OR treadmill-VR OR CAVE OR CAREN) AND Parkinson*.

### 2.3 Eligibility criteria (inclusion/exclusion)

Population: adults with idiopathic PD (clinical diagnosis). Exclusions: atypical parkinsonism; non-PD samples. Intervention: rehabilitation programs explicitly employing VR, classified as immersive (HMD), semi-immersive (multi-projection systems such as CAVE/CAREN), or non-immersive (screen-based with motion capture/force platforms) provided that a virtual scenario is a central therapeutic component. Exclusions: interventions without VR (e.g., conventional physiotherapy only; generic 2D games without VR environments). Outcomes: motor (e.g., BBS, TUG, FGA, gait/posturography parameters, UPDRS/MDS-UPDRS), non-motor (executive functions, MoCA, TMT, QoL/PDQ-39), tolerability (Simulator Sickness Questionnaire-SSQ; adverse events), and adherence/engagement. Study designs: RCTs, quasi-experimental studies, pre–post designs, pilot/feasibility studies. Language: English or Italian. Period. Only articles published 2010–2025 are counted as included studies. Items outside this range or not fully meeting the VR + PD criteria are handled as Contextual evidence and are not counted among the included PD + VR studies.

### 2.4 Operational definitions (VR classification and setting)

For each study we coded: (1) immersion level as immersive (typically head-mounted display-based), semi-immersive (e.g., multi-screen/projection or treadmill-VR systems), or non-immersive (screen-based systems with motion capture/sensors) when a virtual scenario was central to the intervention; (2) setting (lab/hospital vs. home/tele-rehabilitation); (3) dose (number of sessions, minutes per session, weeks, and whether follow-up was reported); (4) control type (sham/yoked, active, or none); and (5) safety/tolerability (adverse events and/or Simulator Sickness Questionnaire [SSQ], where reported). The term exergaming was used descriptively for game-based exercise content and not as a separate category of immersion.

### 2.5 Study selection

All records were de-duplicated using reference management software. Two reviewers independently screened titles/abstracts and subsequently full texts. Disagreements were resolved through discussion and consensus; when consensus was not reached, a third reviewer adjudicated the decision. Inter-rater reliability was not formally quantified (e.g., Cohen’s kappa), as the review adopted an integrative narrative approach; however, independent screening and structured consensus procedures were applied to enhance selection rigor and reduce subjective bias.

Count totals (identified, duplicates removed, screened, full-text assessed, included) are reported in Figure 1: Narrative search flow (2010–2025). Figure 1 reports full-text exclusions by explicit category (total *n* = 83), with each record assigned a single primary exclusion reason to ensure verifiable accounting. When more than one exclusion criterion applied, each record was assigned a single primary reason for exclusion to avoid double counting. Articles that did not fully meet PD + VR 2010–2025 criteria but were informative for rationale/design were moved to Contextual evidence and not counted among the included trials.

### 2.6 Synthesis of results

Although this review adopts an integrative narrative approach, formal risk-of-bias assessment was considered where applicable. Specifically, the Cochrane Risk of Bias 2 (RoB 2) tool was applied to randomized controlled trials (RCTs), and the ROBINS-I tool was considered for non-randomized and quasi-experimental studies. Given the heterogeneity of study designs and reporting, a full domain-level RoB scoring was not feasible for all studies. Therefore, in addition to the structured narrative appraisal, we developed a study-level quality appraisal matrix summarizing key risk-of-bias domains across all included studies (Supplementary Table S1). We conducted a qualitative narrative synthesis by domains: balance/posture, gait/freezing, upper-limb, cognitive/executive, quality of life, and tolerability/adherence. Where clinical/methodological heterogeneity was substantial, effects are reported as associations (not causal). Evidence strength was internally graded as follows: strong = consistent findings across multiple controlled studies with reasonably comparable outcomes and no major overriding methodological concerns; moderate = recurrent positive signals across more than one included study, but limited by small samples, active/non-sham comparators, incomplete blinding, or outcome heterogeneity; weak = limited or inconsistent findings supported by few studies and/or methodologically constrained designs; exploratory = preliminary signals derived mainly from feasibility, pilot, pre–post, or otherwise highly heterogeneous studies, insufficient for stable inference. These categories are narrative interpretive labels used to guide synthesis and should not be interpreted as formal GRADE assessments. Pre-2010 PD mechanistic papers, non-PD samples (e.g., older adults), and non-VR interventions were considered as contextual evidence to clarify rationale/design.

To improve transparency, these narrative evidence-strength labels were assigned at the outcome-domain level rather than at the individual-study level. Judgments were based on the convergence of findings across included studies, taking into account: (1) the number of studies contributing to a given domain; (2) the presence or absence of controlled or randomized designs; (3) the comparability of outcomes and intervention targets; and (4) recurrent methodological limitations, including small samples, active rather than sham/yoked comparators, incomplete blinding, and incomplete safety/tolerability reporting. Domain-level ratings are summarized in Supplementary Table S2 and reflected in the corresponding Results and Discussion sections.

### 2.7 Quality appraisal and risk of bias

Given the integrative scope and mixed study designs, no single Risk-of-Bias tool was uniformly applicable across all included studies. Nonetheless, we applied a structured critical appraisal across prespecified domains: study design and randomization/allocation procedures (for controlled studies); adequacy of comparator conditions, distinguishing active comparators from sham/yoked or no-control designs; assessor blinding; completeness of reporting for dose, outcomes, and follow-up; safety/tolerability reporting, including adverse events and/or cybersickness/SSQ where available; and adherence/feasibility reporting.

For transparency, these domains were summarized across the 15 included PD + VR studies in a structured risk-of-bias overview (Table 1). This table provides a comparative snapshot of the main methodological strengths and limitations across studies, including study design, comparator adequacy, assessor blinding, completeness of reporting, and safety/tolerability assessment.

**Table 1:** Structured methodological overview of the included PD + VR studies.

ID	Study (First author)	Study design	Comparator type	Assessor blinding	Reporting completeness (dose/outcomes)	Safety / tolerability reporting	Narrative methodological appraisal
1	Shen and Mak (29)	Controlled	Active	Unclear	Moderate–High	Not reported	Moderate
2	Shen and Mak (19)	RCT	Active	Partial/unclear	High	Not reported	Moderate
3	de Melo et al. (24)	Controlled	Active	Unclear	Moderate	Not reported	Moderate
4	Feng et al. (27)	Controlled	Active	Unclear	Moderate	Not reported	Moderate
5	Pazzaglia et al. (42)	Controlled	Active	Unclear	Moderate	Not reported	Moderate
6	Pelosin et al. (28)	Controlled (dose comparison)	Active	Unclear	High	Not reported	Moderate
7	Hajebrahimi et al. (40)	RCT	Active	Unclear	Moderate	Not reported	Moderate
8	Kashif et al. (43)	Controlled	Active	Unclear	Moderate	Not reported	Moderate
9	Formica et al. (16)	Pre–post pilot	None	No	Moderate	Not reported	High
10	Gulcan et al. (25)	Controlled	Active	Unclear	Moderate	Not reported	Moderate
11	Bosch-Barceló et al. (45)	Feasibility	None	No	Low–Moderate	SSQ reported	High
12	Kashif et al. (18)	3-arm RCT	Active	Partial/unclear	High	Not reported	Moderate
13	Cancela-Carral et al. (17)	Pre–post feasibility	None	No	Moderate	Not reported	High
14	Ghous et al. (46)	Controlled	Active	Unclear	Moderate	Not reported	Moderate
15	Tariq et al. (31)	Controlled	Active	Unclear	Moderate	Not reported	Moderate

This table summarizes key methodological features of the included studies in narrative form. Formal risk-of-bias assessment is provided separately in Supplementary Table S1.

## 3 Results

Unless otherwise specified, findings in the Results refer to the 15 included PD + VR studies (2010–2025) summarized in Table 2. Mechanistic studies, pre-2010 papers, non-PD samples, and studies not fully meeting the eligibility criteria are discussed only as Contextual/Supporting evidence to clarify rationale, interpretation, or translational relevance, and are not counted among the included studies.

**Table 2:** Included PD + VR studies (2010–2025): characteristics and outcomes.

ID	Study (First author)	Year	Sample	Study design	Immersion level	Device / System	Rehab setting	Dose (sessions×min; weeks)	Comparator	Primary outcomes	Secondary outcomes
1	Shen and Mak (29).	2014	51 PD	Controlled study / active-comparator trial	Semi-immersive	KSD dancing, Smart EquiTest, treadmill	Lab + Home	12 weeks (8 lab + 4 home)	Active (technology-assisted vs. control)	ABC, LOS, GAITRite (gait velocity, stride length)	SLS, balance tests
2	Shen and Mak (19).	2015	51 PD	RCT	Semi-immersive	KSD, Smart EquiTest, treadmill	Lab + Home	12 weeks	Active (vs strength training)	Falls rate (↓ at 3–6 mo)	SLS, postural response latency, gait velocity/stride length
3	de Melo et al. (24).	2018	37 PD	Controlled study	Non-immersive	Xbox Kinect	Lab	12 sessions	Active (treadmill)	6MWT (↑ distance)	Walking speed
4	Feng et al. (27).	2019	28 PD	Controlled study	Immersive	Immersive VR (HMD)	Lab	12 weeks	Active (conventional therapy)	BBS, TUG, MDS-UPDRS III, FGA (all improved > control)	NR
5	Pazzaglia et al. (42).	2020	51 PD	Controlled study	Immersive	Immersive VR (HMD)	Hospital/Lab	6 weeks	Active (conventional rehab)	BBS, DGI (↑)	DASH, SF-36 (QoL ↑)
6	Pelosin et al. (28).	2022	96 PD	Dose-comparison controlled study	Semi-immersive (treadmill-VR)	VR treadmill system	Lab	6 vs. 12 weeks	Active (dose comparison 6 vs. 12)	Gait, falls (12w > 6w)	Cognition
7	Hajebrahimi et al. (40).	2022	23 PD	RCT	Non-immersive	VR exergame + rs-fMRI	Lab	4 weeks	Active (RCT)	Cognitive tests (improved)	rs-fMRI: increased precuneus activity
8	Kashif et al. (43).	2022	44 PD	Controlled study	Mixed / not clearly specified	VR + motor imagery	Lab	12 weeks	Active (vs PT)	MDS-UPDRS III (tremor, rigidity, posture ↑)	NR
9	Formica et al. (16).	2023	31 PD	Pre–post pilot study	Semi-immersive	CAREN system	Lab	24 sessions	Pre–post	Executive functions (↑)	Anxiety, depression (↓); coping (↑)
10	Gulcan et al. (25).	2023	30 PD	Controlled comparative study	Immersive (VR arm); AR comparator reported separately	AR/VR gait training	Lab	6 weeks	Active (AR vs. VR)	MDS-UPDRS III, BBS (↑)	ABC, gait analysis (↑)
11	Bosch-Barceló et al. (45).	2024	4 PD (+4 PT)	Feasibility study	Semi-immersive (treadmill-VR)	Gamified VR treadmill	Lab	3 sessions (feasibility)	None (feasibility)	Usability (SUS), NATU Quest	SSQ
12	Kashif et al. (18).	2024	60 PD	3-arm RCT	Immersive	VR vs. Motor imagery vs. Routine PT	Lab	12 weeks	Active (3-arm RCT)	MDS-UPDRS, BBS (VR > MI/PT)	ABCS
13	Cancela-Carral et al. (17).	2024	12 PD (HY I–III)	Pre–post feasibility study	Immersive	Commercial IVR (rowing/cycling)	Lab	14 weeks	Pre–post	TUG, FTSST, 2-min step (↑)	PDQ-39, UPDRS (QoL ↑)
14	Ghous et al. (46).	2024	28 PD	Controlled study	Immersive	VR exergaming	Lab	8 weeks	Active (vs task-oriented training)	BBS, FGA, TUG (VR > TOT)	NR
15	Tariq et al. (31).	2025	28 PD (HY II–III)	Controlled study	Non-immersive	Screen-based VR (not specified)	Lab	8 weeks; 3×/week	Active (vs TOT)	BBS, FGA, TUG (VR > TOT)	NR

AE = adverse events; ABC/ABCS = Activities-specific Balance Confidence Scale; BBS = Berg Balance Scale; CAREN = Computer Assisted Rehabilitation Environment; DGI = Dynamic Gait Index; FGA = Functional Gait Assessment; FTSST = Five-Times Sit-to-Stand; HMD = head-mounted display; HY = Hoehn–Yahr; IMU = inertial measurement unit; IVR = immersive virtual reality; MDS-UPDRS = Movement Disorder Society–Unified Parkinson’s Disease Rating Scale; MI = motor imagery; NR = not reported; PT = physiotherapy; QoL = quality of life; RS-fMRI = resting-state fMRI; SSQ = Simulator Sickness Questionnaire; TOT = task-oriented training; TUG = Timed Up and Go.

### 3.1 Overview of included PD + VR studies and contextual neurofunctional evidence

#### 3.1.1 Contextual/supporting evidence: neural and perceptual foundations of VR rehabilitation in PD

This subsection summarizes contextual/supporting evidence and is not part of the 15 included PD + VR rehabilitation studies. These studies are discussed to clarify mechanistic plausibility, perceptual constraints, and translational rationale for VR-based rehabilitation in PD.

Two pivotal neuroimaging studies by Wu and colleagues provide foundational insight into the mechanisms underlying motor automaticity in Parkinson’s Disease and support the rationale for VR-based cognitive-motor training paradigms. Firstly, Wu and Hallett (36) conducted an fMRI study on patients with PD performing self-initiated finger sequences. They demonstrated that while PD patients can achieve a form of motor automaticity through training, they engage broader cortical areas compared to healthy controls, particularly premotor and parietal regions, indicating compensatory activation. This highlights the difficulty PD patients have in fully engaging basal ganglia circuits, which are typically critical for automatic movement execution. Wu et al. (37) further investigated what happens when attention is redirected to already-automated tasks. In healthy controls, refocusing attention activated prefrontal and motor association areas but left the striatal pattern unchanged. Conversely, PD patients showed decreased connectivity between the putamen and motor cortex, implying a reversal to a controlled movement pattern. This suggests that in PD, even automated behaviors remain fragile and easily disrupted by cognitive load, a phenomenon directly relevant for VR-based dual-task rehabilitation strategies. These findings strengthen the neurophysiological rationale for combining cognitive and motor training in VR and are consistent with the hypothesis that such interventions may support automaticity through striatal and cortical engagement.

A complementary line of evidence comes from immersive VR studies that explore visuospatial control. A study by Young et al. (38) employed immersive virtual reality to investigate how patients with PD perceive optic flow and adjust their heading during walking. In a virtual hallway where optic flow speed was manipulated across the two walls, patients showed consistent veering patterns, LPD (left-side onset) veered rightward and RPD (right-side onset) veered leftward, consistent with a shifted egocentric reference point (ECRP) toward the side of greater basal ganglia degeneration. Regardless of side of onset, all participants tended to veer away from the faster moving wall, indicating an optic flow equalization strategy. These results suggest that perceptual-motor distortions, such as asymmetric optic flow perception or spatial compression, may influence gait and navigation. This is consistent with the rationale for integrating dynamic visuospatial cues into VR-based rehabilitation protocols and emphasizes the potential relevance of personalized calibration.

Extending the sensorimotor focus to multisensory integration, Ramkhalawansingh et al. (39) showed that aging disrupts optimal visual–vestibular integration during self-motion perception. Older adults, unlike younger controls, overweight visual cues under subtle conflict, suggesting that VR environments for PD should minimize sensory mismatch to prevent maladaptive navigation behaviors.

Finally, neuroimaging and neurophysiological studies suggest that VR-based interventions may engage plasticity-related processes in key neural circuits, although direct causal evidence remains limited. Hajebrahimi et al. (40) reported increased precuneus activity following VR exergaming, together with concurrent improvements in memory and naming; these findings may be consistent with default mode network-related modulation, but should not be interpreted as definitive evidence of a specific network mechanism. Pezzetta et al. (41) showed that error-related mid-frontal theta activity during immersive VR tasks is dopamine-sensitive, suggesting that it may serve as a biomarker of error monitoring and learning potential in PD rather than demonstrating a direct therapeutic dopaminergic effect of VR itself.

#### 3.1.2 Included PD + VR studies: clinical findings and translational signals

This subsection summarizes findings from the 15 included PD + VR studies meeting our eligibility criteria (2010–2025). Where broader mechanistic or translational interpretation is offered, this is explicitly identified as contextual rather than direct evidence from the included studies. Building on these neurophysiological foundations, several randomized controlled trials have assessed the clinical impact of VR interventions on motor and functional outcomes. Shen and Mak (19) conducted an RCT assessing the long-term efficacy of a technology-assisted balance and gait training (BAL) protocol in PD. The BAL group showed significantly fewer fallers at 3, 6 and 15 months post-intervention, with improved stride length and postural responses. In a parallel study, Shen and Mak (29) found that BAL enhanced with augmented feedback led to progressive increases in balance confidence and retention of motor gains up to 12 months. These trials suggest that combining kinetic and visual cues with real-time feedback may facilitate long-term motor learning and self-efficacy.

As contextual/supporting evidence on tolerability, Kim et al. (33) evaluated the safety of 20-min immersive walking sessions in a virtual city using Oculus Rift. No major adverse effects on postural stability or simulator sickness were reported, and PD participants showed increased arousal with reduced stress. This study is not part of the 15 included PD + VR rehabilitation studies, but is cited to contextualize the safety and user-experience profile of immersive VR exposure.

Other interventions have investigated feasibility and physiological benefits. De Melo et al. (24) found that Kinect-based VR training significantly improved walking distance and gait speed in PD, while being more tolerable than treadmill protocols. However, cardiovascular adaptations were less sustained, indicating that VR alone may be insufficient for aerobic conditioning unless combined with endurance-focused elements.

High-intensity VR exercise has also shown promise. Cancela-Carral et al. (17) reported that rowing and cycling in immersive VR improved strength, aerobic capacity, quality of life, and MDS-UPDRS scores in early-to mid-stage PD patients, supporting the feasibility of structured, personalized high-intensity protocols.

Complementary evidence comes from comparative RCTs. Pazzaglia et al. (42) found that a 6-week VR program outperformed conventional therapy in improving balance, gait, and upper-limb function. Similarly, Tariq et al. (31) demonstrated that VR rehabilitation was more effective than task-oriented training for dynamic balance and gait speed.

Some studies have explored hybrid interventions. Kashif et al. (43) showed that combining VR with motor imagery yielded superior improvements in tremor, posture, and bradykinesia compared to physical therapy alone. A later trial (18) confirmed that VR + PT led to stronger and more durable improvements than MI + PT or PT alone, highlighting the neuroplastic potential of immersive, feedback-rich environments.

Among the included PD + VR studies, Pelosin et al. (28) reported that only the 12-week VR training condition (vs. 6 weeks) was associated with improvement in executive function and reduced fall risk. However, this should be interpreted as a preliminary single-study observation rather than as evidence of an established dose–response relationship, pending replication in larger and methodologically comparable studies. As contextual/supporting evidence, Hoang et al. (23) reported reduced DLPFC activation after intensive physical training, and Sasikumar et al. (44) showed that intact working memory predicted better gait adaptation during split-belt treadmill training in PD with freezing of gait; these findings were not derived from the included PD + VR rehabilitation studies but help interpret potential cognitive-motor response mechanisms.

From a cognitive-motor integration perspective, Formica et al. (16) demonstrated that the CAREN system improved executive functions, mood, and coping strategies, con-firming the broader neuropsychological potential of ecologically valid VR interventions.

Among the included PD + VR studies, Bosch-Barceló et al. (45) reported favorable usability and engagement data for a dual-task treadmill-based VR system. As contextual/supporting evidence, Ghous et al. (46) found benefits of non-immersive VR exergaming over task-oriented circuit training in older adults; this study was not part of the included PD + VR evidence base and is cited only to support the broader translational relevance of low-cost adaptive platforms.

Upper-limb function remains underrepresented among the included PD + VR intervention studies. As contextual/supporting evidence, Bissolotti et al. (47) validated a Leap Motion-based serious game platform for manual dexterity assessment in PD, suggesting feasibility and tolerability of technology-assisted upper-limb applications, but this evidence should not be interpreted as part of the included interventional dataset.

#### 3.1.3 Methodological appraisal of included studies

The structured appraisal summarized in Table 1 highlights the recurrent methodological constraints across the included studies. Across the 15 included PD + VR studies, the most recurrent methodological limitations were small sample sizes, frequent use of active rather than sham/yoked comparators, and limited reporting of assessor blinding. Randomized designs were present in several studies, but allocation procedures and blinding details were not always described with sufficient clarity. Safety and tolerability reporting were inconsistent across the 15 included studies. Only a minority explicitly reported safety-related outcomes, and just one study clearly documented SSQ-based cybersickness assessment in Table 2 (45); a few others referred more generally to tolerability or usability, whereas most did not provide structured adverse-event or cybersickness reporting. This limited the comparability of safety findings across studies. Pre–post and feasibility designs contributed useful preliminary signals, particularly for usability, executive outcomes, or high-intensity immersive protocols, but they necessarily reduced confidence in causal interpretation. Overall, the evidence base is clinically promising but methodologically mixed, which is why effect statements in this review are framed as associations rather than definitive efficacy claims.

In addition, Table 2 shows a recurrent presence of “NR (Not Reported)” entries across several domains, particularly for secondary outcomes, safety/tolerability, and in some cases intervention details. Only 1 of the 15 included studies explicitly reported SSQ-based cybersickness assessment, whereas structured adverse-event reporting was absent in most studies. A small number of studies provided general statements on tolerability or usability, but without standardized reporting. This limits the assessment of safety profiles and risk–benefit balance.

This pattern of incomplete reporting has important implications. First, it limits the interpretability of clinical outcomes, because the absence of reported secondary measures (e.g., quality of life, upper-limb function, or detailed gait parameters) reduces the ability to assess the breadth and consistency of intervention effects. Second, insufficient reporting of safety and tolerability prevents meaningful evaluation of risk–benefit balance, which is particularly relevant for VR-based interventions involving sensory stimulation and potential cybersickness. Third, incomplete methodological reporting (e.g., dose specification or follow-up data) reduces reproducibility and limits the applicability of findings to clinical practice.

The prevalence of “NR” entries suggests that the current evidence base is affected not only by methodological heterogeneity and small sample sizes, but also by reporting limitations that constrain both evidence synthesis and clinical translation.

#### 3.1.4 Heterogeneity across included studies and implications for interpretation

The 15 included PD + VR studies were markedly heterogeneous across several clinically relevant dimensions, including immersion level, device type, intervention dose, comparator condition, setting, and outcome selection. Interventions ranged from immersive head-mounted display protocols to semi-immersive treadmill-based systems and non-immersive screen-based or exergaming platforms; dosages varied from brief feasibility exposure over 3 sessions to structured programs lasting up to 14 weeks; comparator conditions ranged from active rehabilitation controls to pre–post or feasibility designs with no control group; and outcomes were distributed across balance, gait, falls, executive functions, quality of life, usability, and tolerability.

This heterogeneity has important implications for interpretation. First, it limits direct cross-study comparability and reduces confidence in any inference that a specific VR modality, immersion level, or dosage is intrinsically superior to another. Second, it means that observed improvements are better interpreted as intervention-specific or protocol-specific signals rather than as evidence of a uniform class effect of “VR rehabilitation” in PD. Third, variability in outcome selection complicates synthesis, because some studies prioritized gait and balance, others focused on cognitive or executive domains, and still others emphasized feasibility, usability, or tolerability.

From a clinical translation perspective, this variability suggests both opportunity and caution. On the one hand, it indicates that VR can be adapted to different rehabilitation goals and delivery formats. On the other hand, it prevents the current evidence base from supporting a standardized prescription regarding optimal immersion level, session intensity, program duration, or comparator-informed efficacy. For this reason, the present review interprets the literature primarily in terms of recurring clinical signals, feasibility, and methodological constraints, rather than as a basis for definitive protocol recommendations. Future studies should aim for greater standardization of reporting, clearer intervention taxonomy, and more comparable outcome sets to improve interpretability and support clinical implementation.

### 3.2 VR for motor rehabilitation in PD

#### 3.2.1 Gait and balance training

Among the included PD + VR studies, the clearest clinical signals concern gait, balance, and functional mobility. Motor impairments, particularly gait dysfunction and postural instability, are among the most debilitating symptoms of Parkinson’s Disease (PD). Patients commonly exhibit shortened step length, increased gait variability, freezing of gait (FOG), and impaired postural control, all of which contribute to limited mobility and increased fall risk. Traditional gait rehabilitation typically targets step initiation, weight shifting, and balance control. However, these protocols often lack engagement and ecological validity, limiting their long-term impact.

Virtual Reality (VR)-based motor rehabilitation addresses these shortcomings by offering immersive, adaptive environments that simulate real-life walking challenges. VR systems incorporate real-time feedback, multisensory integration, and task-specific motor retraining to improve mobility outcomes in PD patients (29, 48–50). VR-enhanced gait training enables progressive exposure to walking scenarios such as obstacle avoidance, terrain variation, and narrow pathways, helping patients improve stride regularity, step length, and dynamic balance (51).

Among the included PD + VR studies, Gulcan et al. (25) reported improvements in postural control and walking confidence, while Feng et al. (27) found that VR-integrated training improved balance and gait-related outcomes compared with conventional therapy. These findings support an association between VR-based rehabilitation and better gait/balance performance within the included evidence base, although heterogeneity in protocols and comparators precludes firm causal inference (52).

A major advantage of VR rehabilitation is the delivery of real-time visual and auditory feedback, which reinforces proper gait mechanics. This feature helps correct gait asymmetries, common in PD due to basal ganglia dysfunction, by encouraging patients to lengthen steps, maintain rhythm, and optimize weight distribution. In addition, controlled perturbation training within VR allows patients to practice postural responses to dynamic challenges (e.g., shifting visual stimuli, virtual obstacles), leading to improved reactive balance control and reduced step hesitation.

Crucially, VR training also incorporates dual-task conditions, proprioceptive feedback, and simulated navigation tasks to reinforce anticipatory and adaptive postural strategies. Within the included studies, this pattern is compatible with improved mobility and fall-related outcomes (18), whereas the broader interpretation regarding compensatory mechanisms and real-world transfer is additionally informed by contextual/supporting literature (53–55).

Beyond biomechanical gains, VR’s interactive, gamified nature increases patient motivation and adherence, mitigating the monotony of conventional therapy. As technology evolves, with wearable motion sensors, AI-driven feedback, and home-based tele-rehabilitation platforms, VR is positioned to play a growing role in fall prevention and motor autonomy in PD (56).

Taken together, the evidence for gait, balance, and functional mobility outcomes was judged as moderate, based on recurrent positive signals across multiple controlled studies, but limited by heterogeneity of protocols, active comparators, and incomplete methodological reporting.

#### 3.2.2 Motor learning and neuroplasticity

This subsection focuses on motor learning and neuroplasticity as mechanistic frameworks for interpreting the motor findings of the included PD + VR studies. It should not be read as direct mechanistic proof derived solely from the 15 included rehabilitation studies. Rather, it offers a hypothesis-informed interpretation of contextual/supporting evidence relevant to gait, balance, postural adaptation, movement refinement, and motor skill retention. Cognitive and executive outcomes are discussed separately in Section 4 to maintain a clear distinction between motor-training mechanisms and the clinical evidence for cognitive rehabilitation.

Rehabilitation-induced neuroplasticity is a central concept in PD motor recovery (57–59). PD disrupts movement initiation, scaling, and automaticity. Adaptive training may therefore support compensatory reorganization within sensorimotor circuits and improve motor performance through repeated practice (57, 58). Within this framework, VR may facilitate motor learning by combining repetition, error correction, augmented feedback, and task-specific training in ecologically relevant environments (19–21).

Unlike conventional rehabilitation, which often relies heavily on explicit instruction, VR may support aspects of implicit motor learning. It allows patients to refine movement execution through repeated sensorimotor interaction. Real-time visual, auditory, and proprioceptive feedback may reinforce step regulation, postural adaptation, weight shifting, and movement timing. In turn, this may support retention of more efficient motor patterns (36, 60–62). These mechanisms are particularly relevant to gait and balance rehabilitation, where repeated adjustment to feedback-rich environments may strengthen movement consistency and motor adaptability (29, 48–51).

A further mechanistic hypothesis is that VR-based motor practice may engage sensorimotor and frontal-motor networks involved in movement planning, monitoring, and adaptation. In this view, task-specific error-based learning and progressive challenge may contribute to improved movement fluidity, stride regularity, and postural transitions (38, 63). However, these mechanisms remain inferential. They should not be interpreted as directly demonstrated mediators of the clinical effects observed in the included intervention studies.

The structured and gamified nature of VR may also enhance repetition, motivation, and adherence. These factors are themselves relevant to motor skill acquisition and retention (24–26, 64–66). Within the included studies, such mechanisms are most plausibly linked to the recurring signals observed for balance, gait, functional mobility, and selected upper-limb outcomes (18, 19, 24, 25, 27, 28, 31, 42). Broader claims regarding executive or cognitive rehabilitation are considered separately in Section 4, where the corresponding clinical evidence is reviewed directly (16, 28, 40).

## 4 VR for cognitive rehabilitation in PD

Whereas Section 3.2.2 addresses motor learning and neuroplasticity mainly as mechanistic frameworks for interpreting motor outcomes, the present section focuses on the clinical evidence for cognitive and executive outcomes associated with VR-based rehabilitation in PD (16, 28, 40).

Evidence for cognitive outcomes in the included PD + VR studies is preliminary, relatively sparse, and more heterogeneous than the evidence for motor outcomes (16, 28, 40). Accordingly, this section distinguishes three types of evidence: VR interventions directly targeting cognitive/executive functions; cognitive or executive changes emerging as secondary effects of primarily motor or motor-cognitive training; and studies primarily designed for motor endpoints in which cognitive measures were included only as secondary or exploratory outcomes. Contextual/supporting literature is discussed separately to interpret plausibility, but is not counted as direct evidence from the included PD + VR studies. The evidence for cognitive and executive outcomes was judged as exploratory to weak, because it derives from a limited number of heterogeneous studies, often with small samples and with cognitive findings reported as secondary rather than primary outcomes.

### 4.1 Direct cognitive/executive training in VR

Among the included PD + VR studies, only a limited subset was designed with a primary emphasis on cognitive or executive outcomes. Formica et al. (16), using the CAREN system, reported improvements in executive functions together with favorable effects on mood and coping strategies. Hajebrahimi et al. (40) also reported gains on selected cognitive tests, alongside rs-fMRI changes, suggesting that some VR-based interventions may directly engage executive/cognitive domains. However, these studies remain few in number, include relatively small samples, and should therefore be interpreted as preliminary rather than definitive evidence of cognitive efficacy.

### 4.2 Secondary cognitive effects of primarily motor or motor-cognitive VR training

A second category includes studies in which cognitive or executive changes emerged as secondary effects of interventions primarily focused on gait, balance, or motor-cognitive training. In this context, Pelosin et al. (28) reported that a longer-duration VR treadmill program (12 weeks vs. 6 weeks) was associated not only with reduced fall risk but also with improvement in executive function, suggesting that cognitive benefits may arise indirectly from sustained dual-task or motor-cognitive training. These findings are clinically relevant, but they should be interpreted as secondary cognitive signals rather than evidence from interventions specifically designed as cognitive rehabilitation protocols (28).

### 4.3 Studies primarily designed for motor endpoints with secondary or exploratory cognitive measures

A third category is only limitedly represented in the included PD + VR literature and refers to studies primarily designed around motor endpoints in which cognitive outcomes were assessed as secondary or exploratory measures. In these cases, cognitive findings are informative but should not be weighted in the same way as results from trials explicitly targeting cognitive rehabilitation. Within the included PD + VR literature, this pattern is less consistently represented than the first two categories, but it still reinforces the impression that cognitive effects are possible, although they remain insufficiently characterized in terms of specificity, reproducibility, and dose–response (16, 28, 40). Therefore, compared with the more consistent motor findings, the evidence for cognitive benefit remains exploratory to weak (16, 28, 40).

The included PD + VR studies suggest that cognitive effects are possible but remain less established than motor outcomes (16, 28, 40). Importantly, these signals do not derive from a single homogeneous evidence base: some studies evaluated cognitive/executive outcomes more directly, whereas others reported them as secondary findings from predominantly motor or dual-task interventions, and still others included cognitive measures only as ancillary outcomes. This distinction is important, because it limits the extent to which the available literature can be interpreted as direct evidence for VR-based cognitive rehabilitation in PD.

## 5 Discussion

The included PD + VR studies suggest that VR-based rehabilitation is associated mainly with improvements in balance, gait-related measures, selected functional outcomes, and preliminary executive/cognitive signals (16, 18, 19, 24, 25, 27, 28, 31, 42). However, the certainty of this evidence remains limited. Samples were generally small, study designs were heterogeneous, and comparators were often active rather than sham-based.

The mechanistic interpretation of these findings, including error-based learning, multisensory integration, and cognitive-motor coupling, is informed partly by the included studies and partly by contextual/supporting evidence (16, 28, 40, 44, 45, 48, 50). These mechanisms should therefore be interpreted as plausible explanatory frameworks rather than conclusively demonstrated mediators of clinical benefit.

Within this framework, VR appears relevant for PD rehabilitation because it can combine motor practice, dual-task demands, and feedback-rich training in a more engaging format than conventional therapy (18, 28, 45, 50). Included studies provide preliminary support for gains in fall-related, executive, and motor outcomes under cognitively demanding conditions. Feasibility studies also suggest favorable engagement and usability (16, 19, 28, 45). At the same time, adherence, safety, and tolerability were not uniformly reported. This limits confidence in real-world generalizability (18, 19, 45). Overall, VR should currently be interpreted as a promising adjunct to conventional rehabilitation rather than a stand-alone or standardized therapeutic replacement (18, 19, 28).

Despite its promise, clinical integration of VR in PD rehabilitation remains incomplete. Adoption in routine care is still limited by accessibility, cost, standardization, technological literacy, and the need for tailored interfaces and support structures for patients with PD (67, 68). This perspective is consistent with Canning et al. (67), who highlighted the importance of translating rehabilitation evidence in PD into scalable, individualized, and clinically implementable care models. Their framework is directly relevant to the present review, because the current PD + VR literature shows promising signals but remains limited by heterogeneity, incomplete standardization, and insufficient real-world implementation data. These barriers must be addressed before VR can be more widely implemented in routine practice.

Our structured critical appraisal identified several recurrent sources of potential bias. These included small samples, predominance of active rather than sham/yoked comparators, infrequent or unclearly reported assessor blinding, and inconsistent reporting of adverse events, cybersickness, and adherence. These limitations do not negate the observed clinical signals. However, they reduce confidence in the magnitude and specificity of the reported effects. Future trials should include sham/yoked feedback, blinded raters, pre-registered protocols, and a core set of motor and patient-reported outcomes. Standardized tolerability measures such as the SSQ should also be incorporated.

A multidisciplinary and personalized approach remains essential, particularly for older adults with PD who face additional socio-technical vulnerabilities (69). Lack of protocol standardization and limited clinician training continue to restrict implementation in everyday practice. These factors also contribute to outcome variability and reduce cross-study comparability. As a result, the current literature does not yet support standardized recommendations regarding optimal immersion level, dosage, or outcome-specific protocol.

Another important limitation is the frequent presence of missing or non-reported data across key domains in Table 2. This affects interpretation of safety, tolerability, and secondary functional outcomes. As a result, the current literature provides a clearer indication of efficacy signals than of complete intervention profiles needed for real-world implementation.

Looking forward, technological innovation may help address some of these limitations. AI-driven VR systems may enable more dynamically adjusted therapies. Tele-rehabilitation platforms may broaden access to home-based care. Biometric monitoring may also improve understanding of engagement and responsiveness in real time (70–72). However, these possibilities remain prospective. Their clinical value still requires confirmation in more robust and standardized studies.

For this reason, we interpreted the evidence using narrative strength categories rather than definitive efficacy labels. Motor findings for gait/balance were judged overall as moderate. Selected upper-limb and quality-of-life findings were judged weak-to-moderate. Cognitive/executive findings were judged exploratory to weak, depending on study design consistency and reporting quality.

### 5.1 Cybersickness, adverse events, and safety considerations

A clinically relevant aspect of VR-based rehabilitation that remains insufficiently addressed in the current literature is safety, including cybersickness and adverse events. Across the 15 included PD + VR studies, reporting of safety and tolerability was inconsistent and often limited. As shown in Table 2 and the structured methodological appraisal (Table 1), only a minority of studies explicitly reported safety-related outcomes, and just one study clearly documented Simulator Sickness Questionnaire (SSQ)-based assessment of cybersickness (45), whereas most studies did not provide structured adverse-event reporting or detailed tolerability data.

This lack of systematic reporting has important implications. First, it limits the ability to evaluate the risk–benefit profile of VR interventions, particularly for immersive systems that may induce visual–vestibular mismatch, dizziness, nausea, or disorientation. Second, it constrains clinical decision-making regarding patient selection, as individuals with advanced disease, postural instability, cognitive impairment, or vestibular sensitivity may be at higher risk of adverse responses. Third, it reduces comparability across studies, preventing identification of protocol-specific safety profiles related to immersion level, session duration, or task complexity.

Available evidence, including contextual/supporting studies, suggests that VR is generally well tolerated in controlled settings, with low incidence of severe adverse events and acceptable levels of simulator discomfort. However, these findings should be interpreted with caution due to limited sample sizes, short exposure durations, and the absence of standardized safety reporting across most included studies.

Additional clinical studies in Parkinson’s disease and neurological rehabilitation help contextualize this issue. Beyond the single included study clearly reporting SSQ-based assessment of cybersickness, additional clinical studies in Parkinson’s disease have systematically examined tolerability in immersive VR using standardized tools. Kim et al. (33) reported no major adverse effects or relevant simulator-sickness worsening after 20-min immersive walking exposure, while Campo-Prieto et al. (73) found no SSQ symptoms after short immersive exergaming sessions in patients with mild-to-moderate Parkinson’s disease. Likewise, Yun et al. (74) observed no significant increase in SSQ scores across a 10-session fully immersive dual-task exergame program, despite good acceptability and satisfaction. Importantly, the more detailed safety monitoring reported by Silva et al. (75) suggests that adverse events may be more frequent than usually documented in Parkinson’s disease VR studies, although most events were mild and SSQ scores remained stable over time. The authors also noted that interpretation of the SSQ in Parkinson’s disease requires caution because several questionnaire items overlap with common non-motor symptoms of the disease. In addition, Bosch-Barceló et al. (45), included in the present review, provided SSQ-based tolerability data in a gamified treadmill-based VR feasibility study. Similar negligible SSQ scores have also been described in immersive stroke rehabilitation by Heinrich et al. (76), supporting the broader neurological rehabilitation literature.

From a clinical perspective, safety considerations should be explicitly integrated into VR rehabilitation protocols. This includes gradual exposure, monitoring of symptoms during sessions, use of standardized tools such as the SSQ, and adaptation of immersion level and task demands to individual patient characteristics. Particular attention should be given to fall risk, fatigue, and sensory overload, especially in older or more vulnerable PD populations.

Future research should prioritize systematic and standardized reporting of adverse events, cybersickness, and tolerability outcomes, including severity, timing, and dropout due to intolerance. This is essential not only for improving internal validity but also for enabling safe and scalable implementation of VR-based rehabilitation in real-world clinical settings.

### 5.2 Clinical implementation considerations

The implementation challenges identified in the present review are broadly aligned with the rehabilitation priorities outlined by Canning et al. (67), particularly regarding individualized prescription, patient selection, feasibility in routine care, and the need to bridge efficacy research with practical delivery models. From a clinical implementation perspective, the VR modalities that currently appear most feasible for routine Parkinson’s disease rehabilitation are semi-immersive and non-immersive systems, including treadmill-based VR, screen-based platforms, and sensor-based exergaming, because these formats have been evaluated in structured rehabilitation settings and, in some cases, in combined lab/home pathways (19, 24, 28, 29, 31). Contextual evidence from other neurological populations, including multiple sclerosis, also supports the potential of VR and exergaming approaches for improving balance outcomes, although such findings should not be directly generalized to PD without condition-specific trials (77). These approaches may be easier to integrate into existing physiotherapy workflows than fully immersive systems, while still providing task-specific feedback and engagement. Fully immersive VR remains promising, particularly for dual-task training, motivation, and high-intensity personalized exercise, but its broader routine implementation may still be constrained by equipment demands, setup complexity, tolerability monitoring, and the need for dedicated staff training (17, 18, 42, 45). This interpretation is also consistent with broader evidence on non-invasive feedback-based rehabilitation approaches in PD, suggesting that feedback, patient engagement, and adaptive training may support both motor and selected cognitive rehabilitation targets (78).

Regarding patient selection, the available studies suggest that the most suitable candidates are patients with mild-to-moderate PD, especially those who retain sufficient mobility and cognitive resources to interact safely with feedback-rich and cognitively demanding environments (17, 28, 31). In particular, individuals in earlier to mid disease stages may benefit most from gait, balance, and dual-task-oriented VR protocols, whereas patients with more advanced disability, marked postural instability, or significant cognitive impairment may require simpler, more supervised, or non-immersive formats (16, 17, 28). Although the current evidence does not yet support strict responder profiling, disease stage, balance status, executive function, fatigue, and digital confidence are likely to be relevant clinical considerations when selecting candidates for VR-based rehabilitation (16, 28).

Several gaps still prevent VR from being adopted as a standard-of-care intervention in PD rehabilitation. Across the included studies, the evidence base remains limited by small sample sizes, methodological heterogeneity, predominance of active rather than sham/yoked comparators, incomplete assessor blinding, and inconsistent reporting of safety, cybersickness, adherence, and dose (18, 19, 28). In practical terms, this means that the current literature provides a clearer picture of efficacy signals than of tolerability profiles, despite tolerability being critical for real-world implementation in PD populations. In addition, the lack of standardized protocols, limited clinician training, cost and accessibility barriers, and the scarcity of real-world implementation and home-based service models continue to restrict broader clinical uptake (68–72). At present, these considerations support VR primarily as an adjunct to conventional rehabilitation rather than a replacement for standard therapy.

Throughout this review, claims regarding future implementation and technological potential are intended as forward-looking and hypothesis-informed perspectives rather than evidence-based predictions, given the moderate-to-low certainty and heterogeneity of the current literature.

## 6 Conclusion

Virtual reality shows promise as an adjunct to conventional rehabilitation in PD, with encouraging associations but heterogeneous methods and small samples limiting certainty. By simulating real-world challenges in immersive, interactive environments, VR enables targeted intervention across the motor, cognitive, and emotional domains affected by PD. This approach may support neuroplasticity-related adaptation, motor learning, and sustained patient engagement, three pillars essential for effective long-term rehabilitation.

Across the 15 included PD + VR studies, the most consistent signals concern balance, gait-related measures, functional mobility, and selected upper-limb or executive outcomes, although methodological heterogeneity and small samples limit certainty. The broader neurophysiological interpretation of these findings, including error-based learning, dual-task facilitation, and enhanced cognitive-motor integration, is supported by contextual literature and should therefore be interpreted as explanatory rather than definitive clinical evidence from the included studies alone.

Nonetheless, several barriers continue to hinder widespread clinical adoption. The lack of standardized VR protocols across healthcare settings, limited clinician training, cost-related constraints, and technological accessibility issues, particularly among older adults or individuals with cognitive decline, remain significant hurdles. Ensuring cultural and psychosocial inclusivity will be essential to promote equitable implementation. Looking ahead, the integration of artificial intelligence, biometric monitoring, and tele-rehabilitation platforms offers promising avenues to optimize the personalization, scalability, and accessibility of VR-based care. Adaptive, home-based VR therapies capable of adjusting to patient progress in real time may broaden current rehabilitation models and support more personalized care pathways. Continued interdisciplinary research and innovation will be critical to fully realize this potential and ensure that VR technologies are aligned with the evolving clinical needs and lived experiences of people with Parkinson’s Disease.
