# Supplementary material for: Advancing rehabilitation in Parkinson’s disease through virtual reality: a narrative review
Source: Front Neurol. 2026 May 14;17:1761459. doi: 10.3389/fneur.2026.1761459 (PMC13215901; doi:10.3389/fneur.2026.1761459)
Supplement: Supplementary file 1 [file Table_1.docx]

| Supplementary Table S1.  Risk-of-bias assessment and structured quality appraisal matrix of included studies (Cochrane RoB 2 and ROBINS-I framework) | | | | | | | | | | |
| --- | --- | --- | --- | --- | --- | --- | --- | --- | --- | --- |
| **Study** | **Year** | **Design** | **Tool** | **Randomization** | **Allocation concealment** | **Assessor blinding** | **Comparator adequacy** | **Reporting completeness** | **Safety / tolerability** | **Overall risk** |
| Shen & Mak | 2014 | Controlled active-comparator trial | ROBINS-I | NA | NA | Unclear | Moderate | Moderate-High | Not reported | **Moderate risk** |
| Shen & Mak | 2015 | RCT | RoB 2 | Low | Unclear | Partial/unclear | Moderate | High | Not reported | **Some concerns** |
| de Melo et al. | 2018 | Controlled study | ROBINS-I | NA | NA | Unclear | Moderate | Moderate | Not reported | **Moderate risk** |
| Feng et al. | 2019 | Controlled study | ROBINS-I | NA | NA | Unclear | Moderate | Moderate | Not reported | **Moderate risk** |
| Pazzaglia et al. | 2020 | Controlled study | ROBINS-I | NA | NA | Unclear | Moderate | Moderate | Not reported | **Moderate risk** |
| Pelosin et al. | 2022 | Dose-comparison controlled study | ROBINS-I | NA | NA | Unclear | Moderate | High | Not reported | **Moderate risk** |
| Hajebrahimi et al. | 2022 | RCT | RoB 2 | Low | Unclear | Unclear | Moderate | Moderate | Not reported | **Some concerns** |
| Kashif et al. | 2022 | Controlled study | ROBINS-I | NA | NA | Unclear | Moderate | Moderate | Not reported | **Moderate risk** |
| Formica et al. | 2023 | Pre–post pilot study | ROBINS-I (adapted) | NA | NA | No | Low/none | Moderate | Not reported | **High risk** |
| Gulcan et al. | 2023 | Controlled comparative study | ROBINS-I | NA | NA | Unclear | Moderate | Moderate | Not reported | **Moderate risk** |
| Bosch‑Barceló et al. | 2024 | Feasibility study | ROBINS-I (adapted) | NA | NA | No | Low/none | Low-Moderate | SSQ reported | **High risk** |
| Kashif et al. | 2024 | 3-arm RCT | RoB 2 | Low | Unclear | Partial/unclear | Moderate | High | Not reported | **Some concerns** |
| Cancela‑Carral et al. | 2024 | Pre–post feasibility study | ROBINS-I (adapted) | NA | NA | No | Low/none | Moderate | Not reported | **High risk** |
| Ghous et al. | 2024 | Controlled study | ROBINS-I | NA | NA | Unclear | Moderate | Moderate | Not reported | **Moderate risk** |
| Tariq et al. | 2025 | Controlled study | ROBINS-I | NA | NA | Unclear | Moderate | Moderate | Not reported | **Moderate risk** |
