# Supplementary material for: Advancing rehabilitation in Parkinson’s disease through virtual reality: a narrative review
Source: Front Neurol. 2026 May 14;17:1761459. doi: 10.3389/fneur.2026.1761459 (PMC13215901; doi:10.3389/fneur.2026.1761459)
Supplement: Supplementary file 2 [file Table_2.docx]

| Supplementary Table S2. Outcome-domain evidence-strength matrix for the included PD+VR studies | | | | | |
| --- | --- | --- | --- | --- | --- |
| **Outcome domain** | **Main contributing studies** | **Design support** | **Consistency of findings** | **Key limitations** | **Narrative evidence strength** |
| Gait / balance / functional mobility | [19], [24], [25], [27], [31], [42] | Multiple controlled studies, some RCTs | Recurrent positive signals | Small samples, active comparators, heterogeneity | Moderate |
| Falls / fall-related outcomes | [19], [28] | Limited controlled evidence | Promising but limited | Few studies, outcome heterogeneity | Weak–moderate |
| Upper-limb outcomes | [42] and selected mixed-motor studies | Limited controlled evidence | Selective positive findings | Underrepresented domain | Weak–moderate |
| Cognitive / executive outcomes | [16], [28], [40] | Small mixed designs | Preliminary and heterogeneous | Small samples, secondary outcomes | Exploratory–weak |
| Quality of life / mood / coping | [16], [17], [42] | Limited mixed-design support | Positive but not uniform | Outcome heterogeneity | Weak–moderate |
| Safety / tolerability / cybersickness | [45] plus limited contextual data | Very sparse direct evidence | Insufficiently reported | Missing safety data, poor comparability | Exploratory |
